# Algorithmic hospital catchment area estimation using label propagation

**DOI:** 10.1186/s12913-022-08127-7

**Published:** 2022-06-27

**Authors:** Robert J. Challen, Gareth J. Griffith, Lucas Lacasa, Krasimira Tsaneva-Atanasova

**Affiliations:** 1 Hub for Quantitative Modelling in Healthcare, University of Exeter, Exeter, UK; 2 Somerset NHS Foundation Trust, Taunton, UK; 3 Bristol Medical School, Population Health Sciences, University of Bristol, Bristol, UK; 4 Medical Research Council Integrative Epidemiology Unit, University of Bristol, Bristol, UK; 5 School of Mathematical Sciences, Queen Mary University of London, London, UK; 6 Instituto de Física Interdisciplinar y Sistemas Complejos (IFISC), Universidad de las Islas Baleares, CSIC-UIB, Palma de Mallorca, Spain; 7 The Alan Turing Institute, British Library, London, UK; 8 Data Science Institute, University of Exeter, Exeter, UK

**Keywords:** Catchment area, Covid-19

## Abstract

**Background:**

Hospital catchment areas define the primary population of a hospital and are central to assessing the potential demand on that hospital, for example, due to infectious disease outbreaks.

**Methods:**

We present a novel algorithm, based on label propagation, for estimating hospital catchment areas, from the capacity of the hospital and demographics of the nearby population, and without requiring any data on hospital activity.

**Results:**

The algorithm is demonstrated to produce a mapping from fine grained geographic regions to larger scale catchment areas, providing contiguous and realistic subdivisions of geographies relating to a single hospital or to a group of hospitals. In validation against an alternative approach predicated on activity data gathered during the COVID-19 outbreak in the UK, the label propagation algorithm is found to have a high level of agreement and perform at a similar level of accuracy.

**Results:**

The algorithm can be used to make estimates of hospital catchment areas in new situations where activity data is not yet available, such as in the early stages of a infections disease outbreak.

**Supplementary Information:**

The online version contains supplementary material available at (10.1186/s12913-022-08127-7).

## Background

During the COVID-19 pandemic, the rapid assessment of the available capacity of a hospital and the potential demand on its services has been important in identifying geographical areas where hospital services are at risk of becoming overwhelmed. Along with epidemic dynamics, residual hospital capacity guides the imposition of public health measures such as social distancing. When assessing the load on a hospital due to COVID-19 the demand may be unevenly distributed in space and rapidly changing in time. Available capacity may be influenced by multiple factors, including staff availability. At the same time there may be fundamental changes to health provision in the acute response of the pandemic, with for example the cancellation of routine operations. In the early epidemic in the UK, for example, there was block booking of private health care providers to assist the NHS [1], and the rapid creation of large scale field hospitals [2]. In previous work we examined the potential for redirecting patients from one region to another to balance the load of health care provision [3] and we have observed this phenomenon as intensive care units reach capacity [4]. When we consider both the change in provision of services and the redistribution of patients, there is a potential need to redefine the demographic and geographic profiles of health care service providers (“catchment areas” and “catchment populations”) [5] to allow for effective planning.

The catchment area or population of a hospital is a broad concept which serves a number of purposes, such as:

- Definition of the primary population of a hospital (and their demographics) for strategic planning purposes [6].
- Definition of higher level organisational structures and collaborative networks [7].
- Identification of areas with under, or over provision of services
- Calculation (and visualisation) of incidence and prevalence of disease from hospital reported statistics (identifying the denominator) [8] and hence admission rates per head of population.
- Preferred routing of patients to hospitals for optimising specific services.

There are two general approaches to modelling catchment areas which we will discuss in detail - activity based or algorithmic approaches. Algorithmic approaches are based solely on regional population counts and hospital capacity. Activity based approaches minimally require data on hospital activity across all the region at an individual level, such as individual patient admission records.

Either of these individual modelling approaches result in a hospital catchment area that is either overlapping or non-overlapping. An overlapping output may reflect the fact that patients may have a choice in the use of the services, and that a range of individually varying predictors influence individuals’ capacity and willingness to adhere to arbitrarily imposed boundaries. It may also reflect a fundamental organisation of the service, for example the networks of critical care [4], in which some activity of a hospital caters directly for the local population, but other activity is conducted supporting other regional hospitals. As such overlapping approaches may better reflect reality, but non-overlapping outputs are often a necessary simplification for secondary analyses, where cross-classification is not specifiable [9]. It is often desirable for secondary analysis that boundaries align with geographical and organisational boundaries, but non-overlapping outputs may result in real world cases being incorrectly assigned to a hospital based on the catchment area, and this will tend to be spatially uneven, clustering at the fringes of the imposed boundaries [10].

The simplest algorithmic approaches involve a measure of the size of a hospital inversely weighted by straight line distance [11]. This can be extended by models which use an analogy to gravity to calculate the potential field of every hospital, based on both capacity (e.g. beds) and demand (e.g. patients) [11–13]. The resulting potentials may be cut off at a specified value, or where they are exceeded by another hospitals potential, to produce either overlapping or non-overlapping fields. Such algorithmic approaches may not respect geographical or existing organisational boundaries, but they can be used to model hypothetical scenarios, such as the impact of creating a new hospital. Further details of the range of different models that have been proposed have been previously published [5, 8].

Activity based models began with the proportional flow, or Norris-Bailey, model [14, 15], and similar techniques developed by Wennberg and Gittelsohn [16]. These examine the proportion of patients from an area visiting a particular hospital versus the proportion of patients in an area who visit any health care provider. An extension of this was recently used to define catchment areas for major injury following acute trauma [17]. More recently modern statistical approaches have been applied to the same basic activity data including k-Means classification [8], Bayesian regression modelling. [6] or Markov multiscale community detection [7, 18], to define hospital catchment areas, and a k-means clustering algorithm to define administrative hospital groups [19]. Whilst arguably providing a more accurate reflection of reality, activity based models are predicated on the availability and currency of activity data, which may exhibit historical or cultural biases. Depending on the purpose of the catchment area such historical bias may or may not be desirable [8].

Estimation of hospital catchment areas is a simplification of a complex logistical and organisational problem. In England, for example, hospital sites are typically grouped into single organisational units (NHS trusts) which report combined activity. Thus a single unit of health-care provision (NHS trust) may have a range of physical locations, not all of which offer the full range of services. ICU provision is often focused in a single hospital in an NHS Trust, whereas acute or step-down beds may be distributed across multiple sites. Some specialist services, such as intensive care, also may be unevenly distributed, and larger units used as "tertiary referral centres" which take in more complex patients from a wider geographical area.

In the early phase of the COVID-19 pandemic, a rapid estimate was needed of the potential demand on intensive care services as a result of observed and forecast infections, in the context of a changing landscape of health service provision. At this point, there was no comparable data with which to drive activity based models, and volatile estimates of hospital capacity. In order to plan provision of additional ventilators and high dependency beds, we needed a model of geographical catchment areas that could be used to translate regional epidemiological models of infections into a prediction of future admissions to individual hospitals, taking into account the regional demographics, and an estimate of the expected level of care the patients would need. Such a catchment area model must interface with existing spatial boundaries implemented in epidemiological models and publicly available demographic estimates, and fulfil the following criteria:

- Allow a clean one way mapping from fine grained geographic regions (e.g. from regional demographic estimates or epidemiological models) to the coarse grained administrative hospital region.
- Provide contiguous and realistic subdivisions of geographies relating to a single hospital or to a hospital group.
- Provide areas that are determined by the capacity of hospital at different levels of care provision, and the size of the local population, or anticipated size of outbreak in the local population.
- Create regions of approximately equal local supply (e.g. beds) and demand (e.g. patients) at boundaries.
- Respect topological constraints in the mapping data, such as large rivers or inlets, such that the overland route to the hospital is accounted for rather than straight line distance.
- Flexible in that it can be recomputed rapidly if the background parameters change, for example, a regional outbreak or provision of additional hospitals, in a way that is not dependant on individual level activity data.

In this work we present a solution we developed for this problem, and introduce a novel algorithmic catchment area model which is specifically designed to meet the needs of the COVID-19 pandemic as described above, but is globally applicable to the situation where we can quantify demand for a resource and a set of point locations that supply that resource, and could be used, for example, in retail. This model is inspired by label propagation techniques used for community detection in networks [20–22]. The paper is presented as follows; firstly we introduce the algorithm, secondly we describe some illustrative examples, and thirdly we qualitatively compare the output of the algorithm to both manually created organisational boundaries, and to observed patient ICU admissions during the first wave of the COVID 19 pandemic.

## Materials and methods

This section consists of 3 parts: a detailed description of the algorithmic catchment area model, a description of the data used to create initial outputs from the model, and a description of initial assessment of the model against available data.

### Algorithm

**Fig. 1:**
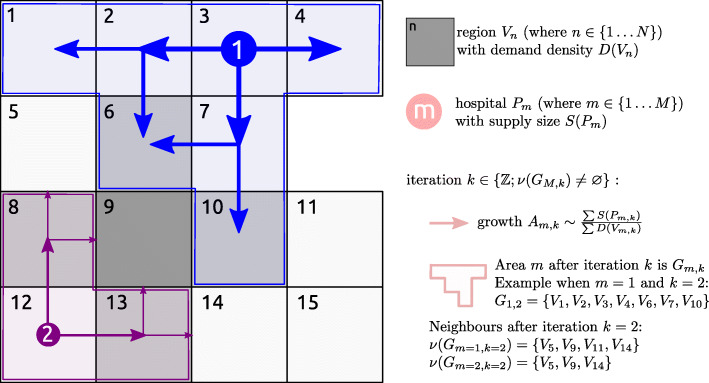
Schematic illustration of the proposed label propagation algorithm. The association of a hospital with a region propagates from the hospital location (P) into the different regions (V) at a rate depending on the hospital capacity S(P) and the population of the region, D(V), at each round of the iteration (k) until there are no more neighbours to propagate a label to. The direction of spread is determined by the geographical neighbourhood of each region V

The algorithm is inspired by label propagation network clustering, where labels correspond to the supply of a service, and the nodes in the network correspond to the demand for the service (see Fig. 1 and Algorithm 1). For illustrative purposes in this paper we will focus on the example of hospitals, where the "supply" is provision of hospital beds, the "demand" is the population size, and the "network" is the neighbourhood of geographical areas under consideration.

To connect supply and demand, or hospital beds to population size, the algorithm propagates a number of labels, each representing the source of supply (e.g. the hospital), through the geographical network, at a rate defined by both the size of the supply (e.g. beds in each hospital), and the demand for the service (e.g. the population) within the areas the label has already propagated to. Thus as demand outstrips supply from a particular source the rate of label propagation associated with that source decreases.

We assume the whole geographical region under consideration can be represented as a mathematical graph, *G* and is divided into *N* smaller regions (parameterisation discussed below), represented by the vertices *V* (where *V*=*V*_*n*_,*n*=1,2,…,*N*) each with known population of size *D*(*V*_*n*_).

We define *M* hospitals located at the geographical points *P* (where *P*=*P*_*m*_,*m*=1,2,3…*M*), and with capacity to supply *S*(*P*_*m*_) beds. Typically there are fewer hospitals than regions (*M*<<*N*). We constrain *P*_*m*_ such that no more than one *P*_*m*_ is found within any given *V*, i.e. each small region hosts no more than one hospital. In practice the assumption that a maximum of one hospital is found in each small region is occasionally not true. When this does happen, we preprocess the data to combine hospitals that are located together into a single entity.

The connections of neighbouring regions of any area *V*_*x*_ are defined by *E*_*x*_=*ν*(*V*_*x*_), and likewise the set of neighbouring vertices of any subgraph *G*_*y*_ are defined by *E*_*y*_=*ν*(*G*_*y*_). These quantities are readily calculated using the geographical intersection of different areas and various algorithms exist to calculate these from geospatial data [23, 24].

Our goal is to divide the graph *G* into *M* labelled sub-graphs *G*_*m*_ such that the sub-graphs are connected, and that neighbouring sub-graphs have similar bed availability per unit population $\left (\frac {\sum S_{m}}{\sum D_{m}}\right)$. We do this by assigning a score for each combination of region and hospital, which is initially zero. For every iteration of the algorithm this score is incremented in any unlabelled region that neighbours a region that has been labelled (i.e. assigned to a specific hospital). The score is increased by a small amount determined by the ratio of supply (hospital beds) available, and demand (population to be served) in the regions assigned to that hospital. Thus labels propagate more quickly from points with a high capacity, through regions with a low population than vice-versa. The first label to propagate to a given area, and for which the score is above a threshold is defined as the “supplier” for that area, which is labelled as such. This ensures that each region is served by only one hospital.


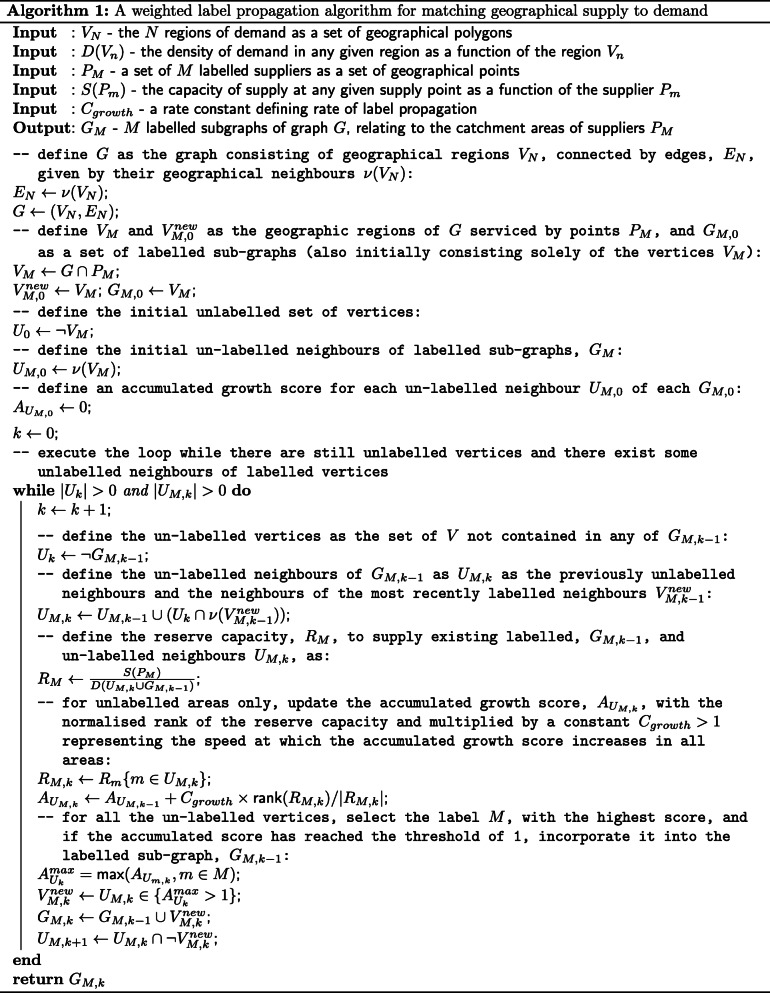


### Qualitative testing data

The algorithm requires firstly an estimate of demand, for this we used population counts, secondly a geographical network and thirdly an estimate of supply, in this case hospital capacity data.

For Great Britain there are detailed estimates of the population at granular geographic detail (lower super output area - LSOA) available from the Office of National Statistics (ONS) for England and Wales, and population estimates by Data Zone (DZ) are provided by the National Records Service (NRS) in Scotland [25, 26]. These population estimates are available by single year of age for each area. These are combined to create a single figure for the adult population of each small geographic area.

Each geographical area is associated with a boundary files for lower super output areas and data zone from the 2011 census, which are provided by the ONS and NRS [27, 28].

To estimate the capacity of hospitals we used a range of primary sources (described in the supplementary materials) to manually compile a list of NHS and independent hospital sites. When not provided in the primary sources, we identified their geographical locations from their postcode, and we estimated bed numbers from both a combination of published NHS statistics and from daily COVID-19 situation reports from early April 2020, provided by the NHS. The situation reports detailed both available beds at this point in time but also gave an indication of maximum surge capacity for high dependency beds. These data were manually curated and are indicative of the state of the NHS at maximal readiness. Bed state estimates for independent hospital providers were also available through the situation reports.

In Northern Ireland, population estimates were not available at a similar geographical resolution as the ONS and NRS sources, and we are unaware of any publicly available hospital capacity estimates. They were therefore not included in this analysis.

The detail of the original data sources we used is presented in the supplementary material, not all of which are publicly available. The algorithm is implemented as an R package arear (available from https://terminological.github.io/arear/), which also contains both the manually curated hospital capacity and data pertaining derived demographics data described here.

### Validation

There is no ground truth for the catchment areas for hospitals in the NHS during the COVID-19 pandemic. The rationale for original development of this algorithm was to make an estimate in absence of any activity data, in the early stages of the pandemic. Since then activity data has become available and this allows us to validate the label propagation approach to the activity based approach.

The activity based mapping takes the form of a many-to-many probabilistic mapping between lower tier local authority districts (LTLA) and NHS Acute Trusts in England derived from Secondary Uses Service (SUS) health-care data for England [29]. We create equivalent probabilistic associations between the coarse grained LTLA and NHS trusts by generating a fine grained lower super output area (LSOA) catchment area for NHS trusts using the label propagation algorithm, and the demographic and bed capacity estimates described above. This is aggregated to coarse grained local authority districts using mapping files provided by the ONS [30], weighted by LSOA population size [25] (Source: Office for National Statistics licensed under the Open Government Licence v.3.0). This equivalent mapping based on the label propagation algorithm is compared to the activity based mapping graphically. To determine the degree of agreement between approaches the expected number of admissions to each NHS trust from each LTLA was estimated using each method. These were compared to each other using the intra-class correlation coefficient [31, 32] using a mean-of-raters, absolute-agreement, two-way random-effects model [33], as implemented in the R package irr [34].

Secondly we obtain the coarse location (partial UK postcode, also known as outcode) from a list of intensive care patients admitted between 20th October 2000 and 16th March 2021 from the CHESS data set [35], which is an anonymised patient level hospital admission data set. We use outcode boundary shapes [36], LSOA demographic estimates, and an areal interpolation [37] to generate an estimate of demographics for each outcode. Using this outcode based regional population estimate, outcode boundary shapes, and the manually curated high dependency unit capacity estimates we calculate an outcode based catchment area estimate from which we are able to predict the NHS trust each patient was admitted to based on their outcode, which we compare to the observed NHS trust from the CHESS data. For this comparison we calculate both the multinomial accuracy, and for each NHS trust, the one-versus-all binomial accuracy as follows:

$$\text{accuracy} = \frac{1}{|X|} \sum_{k \in G} \sum_{g_{obs}(x) = k} I \left(g_{pred}(x) = g_{obs}(x)\right) $$

where *X* is the set of observations, *G* is the set of NHS trusts, *g*_*pred*_ and *g*_*obs*_ are the predicted and observed classes respectively and *I* is the indicator function which returns 1 if the predicted match observed and 0 otherwise.

For the activity based approach we assign each patient to a LTLA by virtue of the geographical location of the centroid of their outcode shape and then determine the most probable NHS trust associated with that LTLA. This forms a prediction of the NHS trust based on the patient’s outcode, which we can compare to the observed NHS trust in the same manner as above.

## Results

### Qualitative testing results

**Fig. 2:**
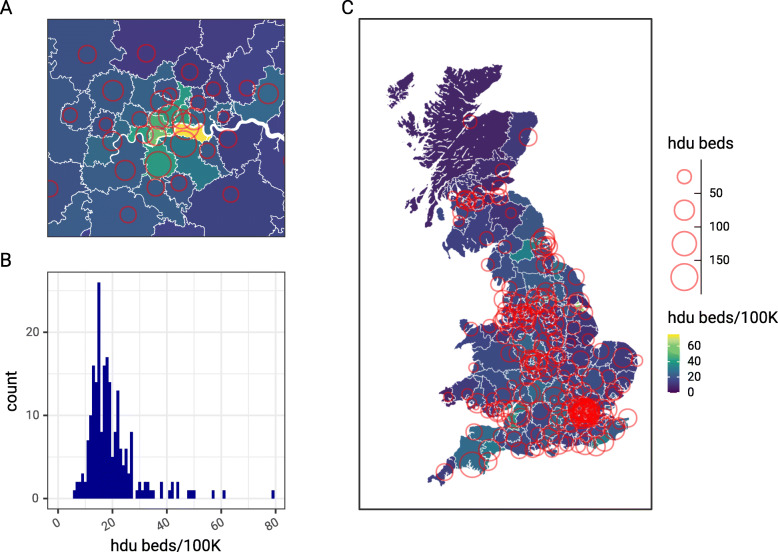
Panels A and C show a LSOA based catchment area map estimated from the high dependency bed state in Great Britain in early April 2020, with catchment area boundaries shown in white. Red circles are NHS hospital sites with size scaled to high dependency bed capacity. Map source: Office for National Statistics licensed under the Open Government Licence v.3.0, Contains OS data Ⓒ Crown copyright and database right 2020. Panel B shows the distribution of high dependency beds per 100K population for each of the catchment areas defined by the algorithm

The results presented in this section qualitatively test the algorithm to determine whether it is producing catchment area regions that are geographically contiguous, aligned with existing demographic boundaries, and respect coarse geographical boundaries such as large rivers. The catchment areas should also produce estimates that minimise differences in the level of service provision from area to area, and we expect the overall regional variation of supply versus demand to be locally smooth. Figure 2 shows a catchment area based on individual hospitals that offered high dependency beds during April 2020, and a regional demand based on population estimates of adults in lower super output areas. The resulting set of catchment areas presented in panel A and C behave as desired in terms of the geographical properties. They also produce a fairly uniform density of high dependency bed provision per capita population, from region to region, as seen in panel B. In areas where there are high densities of hospitals such as London where the algorithm, by design, cannot propagate from centrally located hospitals past more peripheral hospitals, leading to small numbers of areas with high provision per head of population. This is discussed further below.

**Fig. 3:**
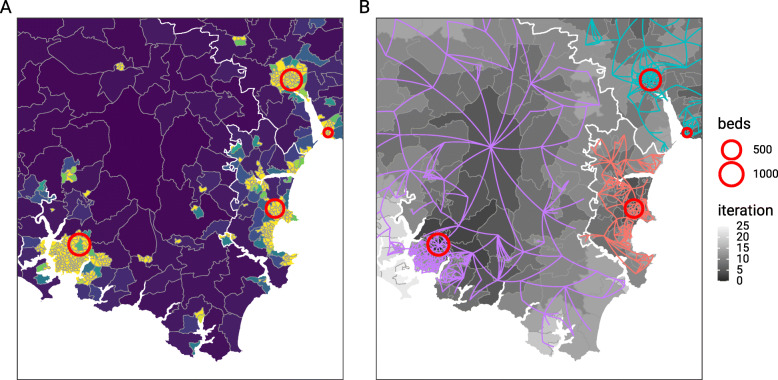
Detail LSOA based catchment area map for NHS trusts estimated from the general hospital bed states in Great Britain in early April 2020. Red circles are NHS hospital sites. In panel A the fill represents a relative measure of regional population density, with yellow areas being high density in and around cities. In Panel B the same areas are shown but this time the fill shows the iteration number at which the algorithm labelled a specific area, and the propagation of the algorithm by arrows. Map source: Office for National Statistics licensed under the Open Government Licence v.3.0, Contains OS data Ⓒ Crown copyright and database right 2020

Further qualitative investigation of the properties of the algorithm are shown in Fig. 3 where we see more regional detail of the same algorithm applied this time to general hospital beds rather than high dependency beds. Panel A shows the boundaries of the estimated catchment areas in white against the population density of a small area of the South West of England containing three hospitals (Plymouth, Torbay and the Royal Devon and Exeter hospitals). We can see in this example the extent of the catchment area to the South of Torbay is defined by the Dart river estuary, thus respecting topological constraints.

Figure 3 panel B shows details about the progression of the algorithm from one iteration to the next, as labels propagate from each of the hospitals into the surrounding areas until encountering another catchment area. As we expect from the design the algorithm is seen to spread from hospital sites quickly through areas of low population (panel A), such as the countryside surrounding Plymouth in the bottom left, and more slowly through areas of higher population such as the areas surrounding Torbay in the middle right.

### Validation

**Fig. 4:**
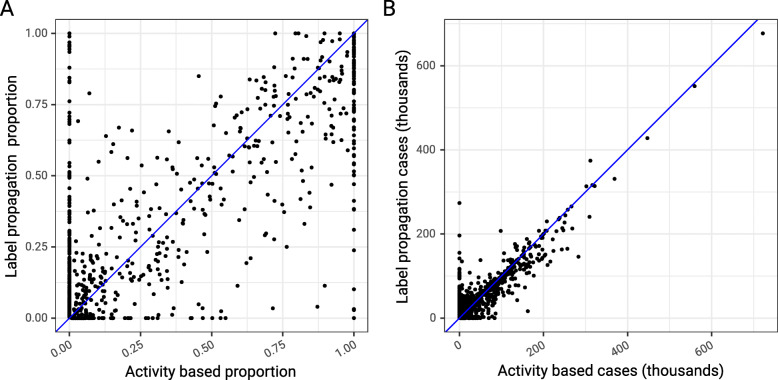
Classification agreement between activity based approach and label propagation algorithm. Each point is a unique combination of lower tier local authority and NHS trust and in panel A the proportion of the LTLA assigned to that trust is plotted for the activity based algorithm on the x-axis and the label propagation algorithm on the y-axis. In panel B the total number of cases assigned to each trust is plotted when the population size for the area is considered. The blue line represents perfect agreement

In comparing the label propagation mapping to the activity based mapping we see that the proportions of any given LTLA that are assigned to any given trust are similar between the two methods Fig. 4, panel A) with a clear trend to agreement. The major differences are seen in the extremes where, for example, in the top left of panel A, the activity based approach may predict that no patients are observed in a given hospital from a given LTLA, whereas the label propagation approach predicts the opposite. Panel B shows the same relationship but this time scaled by the population size in each area, and this shows that the impact of differences between predictions seen in panel A is in areas with smaller populations and is therefore attenuated. Calculation of the intra-class correlation coefficient between the predicted number of cases from each method gives excellent agreement between the two methods, with a value of 0.94 (95% CI: 0.93 – 0.95) using a mean-of-raters, absolute-agreement, two-way random-effects model [33].

**Fig. 5:**
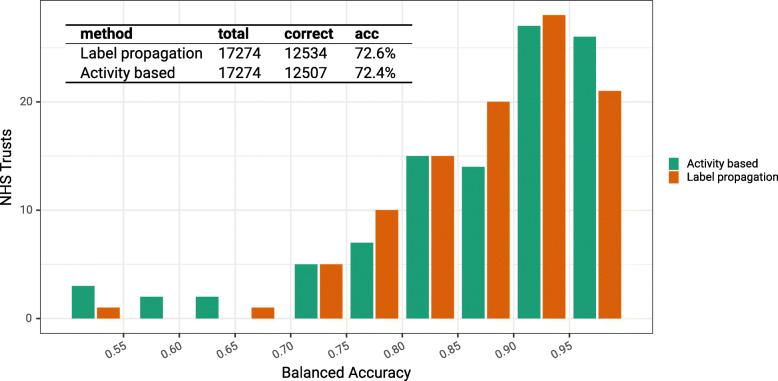
Accuracy measures for the predictions of activity based and label propagation approaches based on UK postcode outcodes, and a subset of observed NHS trust of intensive care admissions in England between 20th October 2000 and 16th March 2021. The histogram shows the distribution of the balanced accuracy for each NHS trust in a one-vs-all binomial evaluation, and the inset table shows the overall accuracy from the multinomial evaluation, along with the raw counts of overall evaluations and correct predictions for each method

In Fig. 5 we compare observed admissions to intensive treatment units (ITU) to predictions made by the label propagation algorithm and the activity based approach. As there are 178 trusts under consideration which form a large number of distractors for each prediction, a low value for the multinomial accuracy could be expected. The overall accuracy of both methods is comparable at 72.6% (label propagation) versus 72.4% (activity based). The distribution of the binomial one-versus-all accuracy in the histogram shows that the prediction performance is better for some trusts than others, and that the accuracy of the activity based approach has greater variability than that of the label propagation approach. Across the whole country exact agreement between the observed location of hospital admission and the predicted location of hospital admission based on the label propagation catchment area was seen in 12534 out of 17274 cases, and the Matthew’s correlation coefficient was 0.72.

The ten NHS Trusts that have the highest number of ITU patients that the label propagation algorithm predicted to be admitted elsewhere, represent 1833 (38.7%) of the total misclassifications. On inspection the majority of these 10 hospitals are major tertiary referral intensive care units, or specialist centres and are in the top quintile of NHS trusts by ITU bed capacity. This could be explained if severely ill patients may end up in specialist centres rather than their closest hospital for treatment, or that in the event of a large surge in cases, patients may overflow from smaller to larger intensive care units. Both of these could lead to misclassification of these patients by the label propagation algorithm.

## Discussion

We have presented an algorithm for rapidly estimating hospital catchment areas for use when activity data is not available. We demonstrate how the output responds to the different capacities of the different levels of care provided (e.g. high dependency versus general hospital beds). We present catchment areas calculated using population size as demand, and total hospital beds as supply. This algorithm may be useful for longer term strategic planning, but was conceptualised as part of an acute response to COVID-19 outbreak. In this case we can use the different parameters for demand, for example local COVID-19 infection prevalence, and different parameters for supply, for example availability to staffed hospital beds. Our approach is novel in that it allows adaptation of local service provision to predictions of disease prevalence from epidemiological models of COVID-19 and real time bed states provided by NHS trusts. This allows us to model the degree of elasticity in the system to absorb localised shocks, caused by regional outbreaks, it helps us to develop a better concept of when services are being at risk of becoming overwhelmed, and allow routing of new admissions away from overloaded hospitals.

Bench-marking our algorithm against activity based approaches produced good to excellent agreement and application to both methods to real world patient admission data produces a very similar result. The finding that naive application of our algorithm to real world patient admission classifies only 72.6% correctly is explained by two things, firstly accuracy at boundaries decreases as the number of boundaries increases [10] and secondly the fact that many of the top 10 misclassified trusts are major tertiary referral centres, which may take patients from distant regions for specialist care. This suggests that our constraint that catchment areas should be non overlapping is not borne out in reality for these cases.

Overlapping catchment areas could be modelled by multiple layers of non-overlapping catchment areas. When we consider the provision of intensive care services in the UK during COVID-19, we propose there are at least 3 layers of hospital service provision: there is a local service, which provides care for patients from nearby. A subset of hospitals additionally provide a regional, or tertiary referral, service layer which takes sicker patients from neighbouring hospitals in larger areas. The final layer is a crisis overflow layer provided by the NHS Nightingale field hospitals [2]. Each of these layers may be considered to have somewhat independent catchment areas, a phenomenon that has been observed in variable uptake of spinal services in Switzerland [38]. We propose that dividing the larger hospitals into local and regional services and considering the tertiary referral network as a second layer, with its own larger catchment area would improve the performance of the algorithm against real activity data. In such a layered model of catchment areas there is interplay between local layer demand for hospital beds and capacity for regional tertiary care provision, which will dynamically affect the “catchment area” for regional tertiary care provision, potentially on a day to day basis. In previous work we looked at the opportunities for balancing the load between different hospitals [3] when transferring COVID-19 patients away from overloaded areas, however moving unwell patients between hospitals is ideally minimised. With this algorithm we enable the dynamic re-specification of local service catchment areas and hospital tertiary referral networks, based on evolving demand. Coupled with flexible load sharing has interesting potential to model or influence patient admissions around the whole hospital network.

Hospital capacity is difficult to accurately estimate. During this work we encountered many of the uncertainties that influence capacity. The ability of a hospital to provide a bed to a patient depends on a multitude of factors, including staff availability, which may vary during the different stages of the pandemic. The ability of hospitals to absorb large numbers of emergency patients by re-configuring their service provision (e.g cancelling routine operations) and providing overflow or "surge" high dependency capacity for short periods of time makes putting a single number on hospital capacity difficult. The ability to recalculate catchment areas based on changing assumptions around capacity is a strength of our approach, and in the future could be used to analyse the impact of introducing new capacity into the hospital system. One further limitation to note is that the algorithm does not consider travel time between regions which may increase both as the geographical size increases but also as the population density increases due to traffic and form a barrier to patients accessing services. Adding a travel time penalty to the rate of label spread into the model is possible given some estimate of the ease of transport within and between regions, and this is an area of future work.

Our approach is limited to situations where the relationship between hospitals and the population is mostly driven by population density and geography. This may be more relevant to national health services such as the NHS, and less relevant to more commercial hospital systems in which there are many different factors influencing the patients decision as to what hospital they attend. However, even within these systems our approach may be useful for service planning in larger hospital groups, when we consider capacity planning for specific services such as radiology.

There are opportunities to extend our algorithm. The general approach of label propagation in networks has been more widely studied and newer approaches described [21, 39, 40] which allow overlapping communities. This may address some of the issues described above. These are appealing and a possible avenue for future extension of the algorithm. There persists however an open question about whether the overlapping nature of hospital service provision observed in activity data is not really a reflection of patient choice, but actually the result of subtly different services, or different levels of service, being provided by different hospitals to different catchment areas. Thus a specialist cancer hospital close to a specialist paediatric hospital will have geographically overlapping catchment areas, but in reality these hospitals are not providing the same service to the same population. This line of argument suggests that the concept of a single overlapping hospital catchment area is also an over-simplification, and when we take into account the heterogeneity of different services offered by a hospital, we propose that a hospital’s overall catchment area may be well modelled by a collection of non-overlapping catchment layers.

## Conclusions

This label propagation algorithm for estimating hospital catchment areas is a pragmatic solution to determining geographical and demographic subsets of the population when there is no previous activity data available. It suits situations where the level of service provision and demand on the hospital system is dynamic, as has been the case in the COVID-19 pandemic. The algorithm is simple and satisfies the major criteria we set out in the introduction, in that it provides a mapping from low level geographic regions which provide contiguous and realistic subdivisions of geographies relating to a single hospital or to a group of hospitals. The areas are determined by the capacity of the hospital and the size of local population, and are approximately equal in terms of local supply (e.g. beds) and demand (e.g. patients) at boundaries.

The algorithm depends solely on data reflecting supply and geographical demand for a service, and as such is quite generic and potentially more widely applicable outside of healthcare. Although we have discussed catchment areas in terms of the capacity of hospital beds, and demand of local populations, there is nothing to prevent us defining capacity in any other way - a heuristic on staffing levels may be appropriate, or in different contexts, availability of medical imaging devices. Likewise, demand may be refined to reflect sub-populations at risk of disease, or may even be the output of a predictive model. As such our approach is applicable to a wide variety of problems.

## Supplementary Information

**Additional file 1** Supplementary material - algorithmic hospital catchment area estimation using label propagation. Sources of hospital capacity data, population estimates, and some additional visualisations.

**Additional file 2** Supplementary data - surge capacity estimates. A curated data set of estimated acute and ITU bed capacity in the NHS and private hospitals at the start of the pandemic, in England, Wales, and Scotland.

## Data Availability

The majority of data and a reference implementation of the algorithm is implemented as an R package arear (available from https://terminological.github.io/arear/). The CHESS data that support part of the validation findings of this study are available from Public Health England but due to the fact the data is at single individual level, albeit anonymised, restrictions apply to the availability of these data. These which were used under license for the current study, and so are not publicly available. This validation data are however available from the authors upon reasonable request and with permission of Public Health England.
