# Supplementary material for: Algorithmic hospital catchment area estimation using label propagation
Source: BMC Health Serv Res. 2022 Jun 27;22:828. doi: 10.1186/s12913-022-08127-7 (PMC9235278; doi:10.1186/s12913-022-08127-7)
Supplement: Supplementary file 1 — Additional file 1 Supplementary material - algorithmic hospital catchment area estimation using label propagation. Sources of hospital capacity data, population estimates, and some additional visualisations. [file 12913_2022_8127_MOESM1_ESM.pdf]

Supplementary material for Algorithmic hospital catchment area estimation using label propagation

Robert Challen (corresponding author - rc538@exeter.ac.uk)[1,2]; Gareth Griffith[3,4]; Lucas Lacasa[5,6]; Krasimira Tsaneva-Atanasova[1,7,8];

1) EPSRC Hub for Quantitative Modelling in Healthcare, University of Exeter, Exeter, Devon, UK.
2) Somerset NHS Foundation Trust, Taunton, Somerset, UK.
3) Bristol Medical School, Population Health Sciences, University of Bristol, Bristol, BS8 2BN, UK
4) Medical Research Council Integrative Epidemiology Unit, University of Bristol, Bristol, BS8 2BN, UK
5) School of Mathematical Sciences, Queen Mary University of London, London E1 4NS, UK
6) Instituto de Física Interdisciplinar y Sistemas Complejos (IFISC) (CSIC-UIB), Campus UIB, 07122, Palma de Mallorca, Spain
7) The Alan Turing Institute, British Library, 96 Euston Rd, London NW1 2DB, UK.
8) Data Science Institute, College of Engineering, Mathematics and Physical Sciences, University of Exeter, Exeter, UK.

Supplementary materials

The algorithm requires firstly an estimate of demand, for this we used population counts, secondly a geographical network and thirdly an estimate of supply, in this case hospital capacity data.

Estimating population density in the United Kingdom during the COVID-19 pandemic

- https://www.ons.gov.uk/peoplepopulationandcommunity/populationandmigration/populationestimates/datasets/lowersuperoutputareamidyearpopulationestimates
- https://www.nrscotland.gov.uk/statistics-and-data/statistics/statistics-by-theme/population/population-estimates/2011-based-special-area-population-estimates/small-area-population-estimates/time-series#2018
- https://www.opendatani.gov.uk/dataset/3333626e-b96e-4b90-82fb-474c6c03b868/resource/64bd8dc4-935f-4bdd-9232-90ff33f24732/

Geographical network

- LSOA11: https://geoportal.statistics.gov.uk/datasets/lower-layer-super-output-areas-december-2011-boundaries-ew-bgc
- DZ11: https://data.gov.uk/dataset/ab9f1f20-3b7f-4efa-9bd2-239acf63b540/data-zone-boundaries-2011
- LGD12: https://data.gov.uk/dataset/05f72866-b72b-476a-b6f3-57bd4a768674/osni-open-data-largescale-boundaries-local-government-districts-2012

Estimating surge hospital capacity in Britain during the COVID-19 pandemic

Identifying a set of capacity data for the NHS proved complex. After several attempts to integrate data from various sources, we ultimately performed a manual curation of the sources listed below, with gaps or inconsistencies filled in by consultation with the relevant hospital's website. The resulting list is a snapshot in time of capacity and not representative of up to date practice. During the course of the COVID-19 pandemic a small number of NHS trusts merged which had to be manually adjusted for. There are also significant limitations due to the different ways the devolved administrations of the UK (England, Wales, Scotland and Northern Ireland) reported situation report of bed capacity during the pandemic, which meant only England and Wales hospitals has assessments of surge capacity, and we had no reliable information about Northern Ireland at all, and hence it was excluded. This does not significantly alter our conclusions here about the nature of the algorithm, but should be borne in mind, if the data set is to be used for other purposes.

NHS and Trust GIS locations (England):

- https://www.nhs.uk/about-us/nhs-website-datasets/
- Lists of independent and NHS hospitals and trusts with location data
- public

NHS Trusts (England)

- https://www.nhs.uk/ServiceDirectories/Pages/NHSTrustListing.aspx
- Lists of NHS trusts and locations (as postcode) with information about services offered and hospital sites
- public

Beds open - NHS England:

- https://www.england.nhs.uk/statistics/statistical-work-areas/bed-availability-and-occupancy/bed-data-overnight/
- https://www.england.nhs.uk/statistics/statistical-work-areas/bed-availability-and-occupancy/bed-data-day-only/
- Information at an NHS trusts level on hospital beds and icu beds available
- public

Critical care capacity in England (pre-pandemic):

- https://www.england.nhs.uk/statistics/statistical-work-areas/critical-care-capacity/critical-care-bed-capacity-and-urgent-operations-cancelled-2019-20-data/
- Prepandemic NHS trust bed and ICU capacity
- public

Wales:

Average daily beds by site:

- https://statswales.gov.wales/v/Hg4K
- Prepandemic ICU and general bed availability
- public

Scotland:

Annual trends in available beds:

- https://www.isdscotland.org/Health-Topics/Hospital-Care/Publications/data-tables2017.asp?id=2494#2494
- Prepandemic Hospital and ICU bed capacity
- public

Sitrep (Situation reports) data:

England:

- filename: Covid sitrep report incl CIC 20200408 FINAL.xlsx
- Acute and ICU beds available in England at site level
- ICU (SIT032) and HDU (SIT033) beds available - many data quality issues and missing trusts
- restricted

Wales:

- filename: NHSWalesCovid19Sitrep-20200408.csv
- Acute and ICU beds available in Wales
- restricted

N.B. No sitrep data for Scotland or for Northern Ireland

Supplementary results

Characterisation of misclassification

In Supplementary Table 1 we qualitatively examine the ten NHS Trusts that have the highest number of ITU patients that the label propagation algorithm predicted to be admitted elsewhere, and mis-classified them. These represent 1833 (38.7%) of the total mis-classifications. The majority of these 10 hospitals are major tertiary referral intensive care units, or specialist centres, as demonstrated by them being in the top quintile of NHS trusts by ITU bed capacity. This result is consistent with both the possibilities that severely ill patients may end up in specialist centres rather than their closest hospital for treatment, or that in the event of a large surge in cases, patients may overflow from smaller to larger intensive care units. Both of these could lead to mis-classification of these patients by the label propagation algorithm, as we see here.

Supplementary Table 1: The NHS trusts with the ten most misclassified covid ITU cases as assigned by the label propagation algorithm

trustId	Trust	April 2020 ITU beds (Centile)	Classification errors
RJ7	St George's University Hospitals Nhs Foundation Trust	147 (91%)	282
R0A	Manchester University Nhs Foundation Trust	151 (93%)	272
RGT	Cambridge University Hospitals Nhs Foundation Trust	90 (73%)	233
RJ1	Guy's And St Thomas' Nhs Foundation Trust	159 (94%)	206
R1K	London North West University Healthcare Nhs Trust	100 (77%)	188
RTD	The Newcastle Upon Tyne Hospitals Nhs Foundation Trust	128 (85%)	178
RBK	Walsall Healthcare Nhs Trust	47 (40%)	138
RYJ	Imperial College Healthcare Nhs Trust	148 (92%)	131
RFF	Barnsley Hospital Nhs Foundation Trust	25 (14%)	103
RJZ	King's College Hospital Nhs Foundation Trust	190 (97%)	102

In Supplementary Table 2 we look at the trusts where there are fewest cases incorrectly assigned to other trusts by the label propagation algorithm. Although these are generally the smaller intensive care units this is not globally the case. This is just a measure of type 1 error and could be the result of an inappropriately large catchment area.

Supplementary Table 2: The NHS trusts with the ten least misclassified covid ITU cases as assigned by the label propagation algorithm

trustId	Trust	April 2020 ITU beds (Centile)	Classification errors
RTR	South Tees Hospitals Nhs Foundation Trust	76 (64%)	21
RTE	Gloucestershire Hospitals Nhs Foundation Trust	187 (96%)	20
RHQ	Sheffield Teaching Hospitals Nhs Foundation Trust	48 (41%)	19
RK9	University Hospitals Plymouth Nhs Trust	139 (88%)	19
RWH	East And North Hertfordshire Nhs Trust	54 (49%)	19
RCF	Airedale Nhs Foundation Trust	16 (5%)	17
RYR	Western Sussex Hospitals Nhs Foundation Trust	54 (49%)	14
RXC	East Sussex Healthcare Nhs Trust	113 (81%)	12
RXL	Blackpool Teaching Hospitals Nhs Foundation Trust	55 (52%)	12
RTG	University Hospital Of Derby And Burton Nhs Foundation Trust	142 (89%)	10

The distribution of patients who attended hospitals with the fewest misclassication errors is shown in Supplementary Figure 1 and these tends to be the intensive care units with fewest attendees, with a few long distance out of area patients.

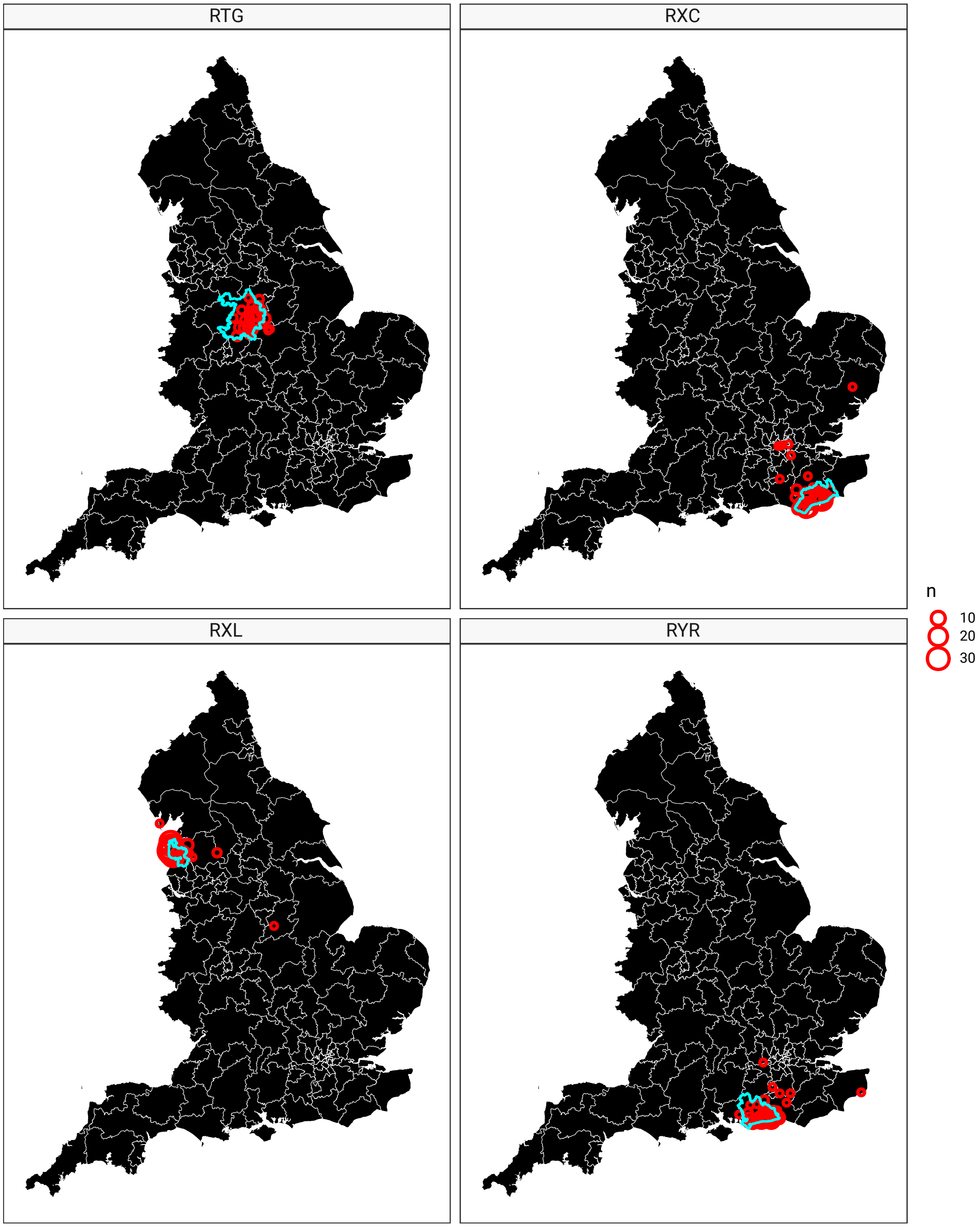

Supplementary Figure 1: The origin of patients attendind the hospitals which are best predicted by the label propagation algorithm. Hospital codes are given in the associated tables. Red circles are patients admitted to the given hospitals and cyan areas the predicted catchment area

The distribution of patients who attended hospitals with the most misclassification errors is shown in

Supplementary Figure 2 and these tends to be the intensive care units with many attendees, spread over much wider areas than the algorithm predicts. These are typically large intensive care units based in dense towns, where there are many other hospitals. A limitation of the label propagation algorithm is that as tertiary referral centres, these hospitals catchment areas for ITU services are probably different in nature from those of the surrounding smaller hospitals. In this case a two-layered approach to the catchment area may be more appropriate, where one layer considers wider tertiary referral and the other locally directly admitted patients who will tend ot be more local.

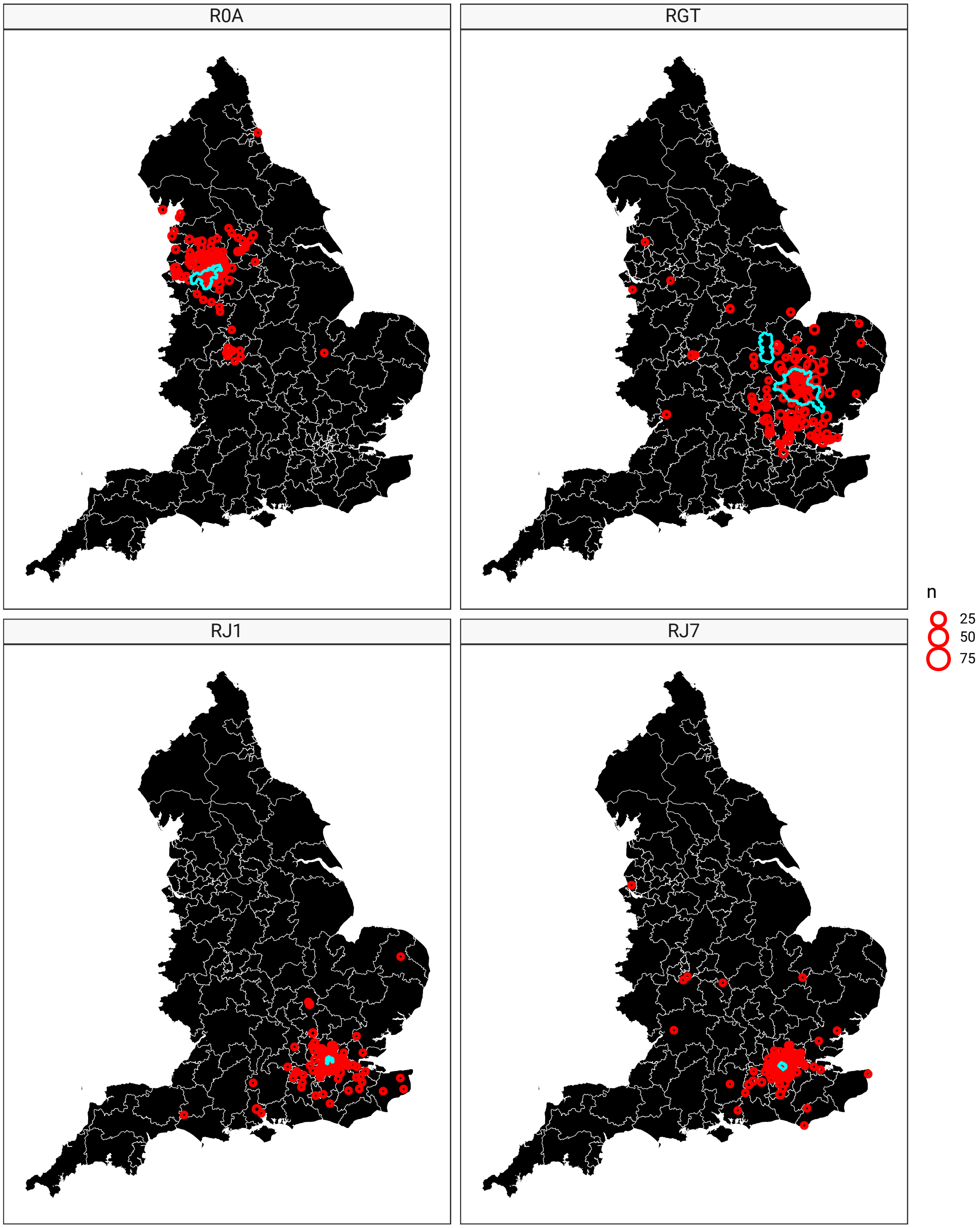

Supplementary Figure 2: The origin of patients attendind the 4 hospitals which are worst predicted by the label propagation algorithm. Hospital codes are given in the associated tables. Red circles are patients admitted to the given hospitals and cyan areas the predicted catchment area
